# The Mediating Effect of Life Satisfaction on Relation between Perceived Physical Attractiveness and Health-Promoting Lifestyle in Korean Adults

**DOI:** 10.3390/ijerph18157784

**Published:** 2021-07-22

**Authors:** Hye-Ryoung Kim, Hwa-Mi Yang

**Affiliations:** 1 College of Nursing, Shinhan University, Dongducheon-si 11340, Korea; hrkim@shinhan.ac.kr; 2 Department of Nursing, Daejin University, Pocheon-si 11159, Korea

**Keywords:** health behavior, lifestyle, satisfaction, appearance, mediator

## Abstract

Physical attraction, perceived in terms of satisfaction with body image and interpersonal relationships, can be an essential factor in an individual’s emotional and social well-being. However, empirical studies that link physical attraction to health are limited. This study explores the mediating effect of life satisfaction on the relation between perceived physical attractiveness and lifestyle of health, making an effort to reach an optimal state in physical, emotional, social, spiritual, and intellectual domains in Korean adults. Four hundred fifty-nine adults in a community setting participated in this cross-sectional study. The results showed positive associations among physical attractiveness, life satisfaction, and health-promoting lifestyle after adjusting for age, gender, marital status, education, and job status. Life satisfaction partially mediated the relationship between perceived physical attractiveness and a higher lifestyle of health (z = 2.80, *p* = 0.005). For health promotion and disease prevention, positive psychology can be a suitable strategy. Physical attractiveness and life satisfaction can be important keys to maintain health-promoting lifestyle behavior.

## 1. Introduction

Craving for a beautiful appearance regardless of age can be a general trend in various societies and has been the subject of ongoing interest in socio-cultural contexts and interpersonal relationships [1,2,3]. Physical attraction, perceived in terms of satisfaction with body image and interpersonal relationships, can be an essential factor in an individual’s emotional and social well-being [4]. In a socio-cultural context that emphasizes beauty, an attractive appearance can be an asset [5,6].

One of the social myths about appearance is that a physically attractive person is more successful, reliable, and competent [7]. Many Korean proverbs reflect this. For example, what looks good, tastes good; the brighter scarlet, the better for the skirt; a beautiful face guarantees a warm heart. As such, physical attractiveness acts as a significant clue in evaluating the person.

An attractive appearance affects confidence and social relations due to the halo effect, a cognitive bias in which overall impressions are judged well by physical attractiveness [4]. In this way, perceived physical attractiveness positively affects psychological well-being [4]. Moreover, there is a report that perception of physical attraction is also related to self-esteem, self-confidence, and consequently, health-promoting behavior [6].

However, empirical studies that link physical attraction to health are limited. Until now, some studies have reported that physical attractiveness is an indicator of health or reproductive status based on body size or symmetry from an evolutionary point of view [8,9]. Other studies have reported a negative correlation between physical attraction and psychological states, such as pain perception or depression [4]. Even though a study investigating 325 female adults in Korea reported that higher perceived physical attractiveness was associated with health-promoting behaviors in the mental and physical domain [6], no study has considered various aspects of a healthy lifestyle, including emotional, social, spiritual, and intellectual domains.

The perception of physical attractiveness leads to satisfaction with body image, and such body satisfaction affects emotional and psychological well-being as a prerequisite for life satisfaction. [10,11,12]. A study of healthy individuals over the age of 50 in Spain found that perceived physical attraction had a strong relationship with life satisfaction [13].

Life satisfaction is the vital precursor of psychological well-being and mental health [11]. Moreover, lower life satisfaction is associated with a higher mortality rate of cardiovascular disease, healthcare service utilization, and costs [14,15]. On the other hand, the higher the life satisfaction, the more that preventive healthcare services are used [16].

Life satisfaction is a health asset from positive psychology [5]. Positive psychological health assets have a prospective association with good health status measured in various ways. For example, positive emotions accelerate cardiovascular recovery after a stressful event [5]. Moreover, reinforcing of positive self-awareness and emphasizing strengths or positive traits rather than negative characteristics have improved depression or psychological well-being [17].

According to the World Health Organization, physical, mental, and social well-being are interconnected components for complete health. Health assets from positive psychology are positive emotions, life satisfaction, optimism, self-regulation, vitality and zest, life meaning and purpose, helping others and volunteering, good social relationships, spirituality, and religiosity [5]. Positive psychological health assets have a prospective association with good health status measured in various ways. For example, inducing positive emotions accelerates cardiovascular recovery after a stressful event [5]. In this way, positive psychology focuses on the experience of positive emotions. Positive psychology has improved depression or psychological well-being by reinforcing positive self-awareness focusing on strengths or positive traits rather than negative characteristics [17].

Among positive psychological health assets, optimism is a well-studied health-related topic [5]. Meanwhile, life satisfaction is relatively unknown with regard to its role in health and the mechanism of inducing healthy behaviors [5]. In addition, it is not easy to find a study that confirmed the relationship between the perception of physical attractiveness and lifestyle behaviors related to health.

This study assumes and intends to investigate that the perception of physical attractiveness affects life satisfaction, health-promoting lifestyle behaviors, and life satisfaction, a positive psychological factor that mediates subjective physical attractiveness and lifestyle behaviors related to health.

**Figure 1:**
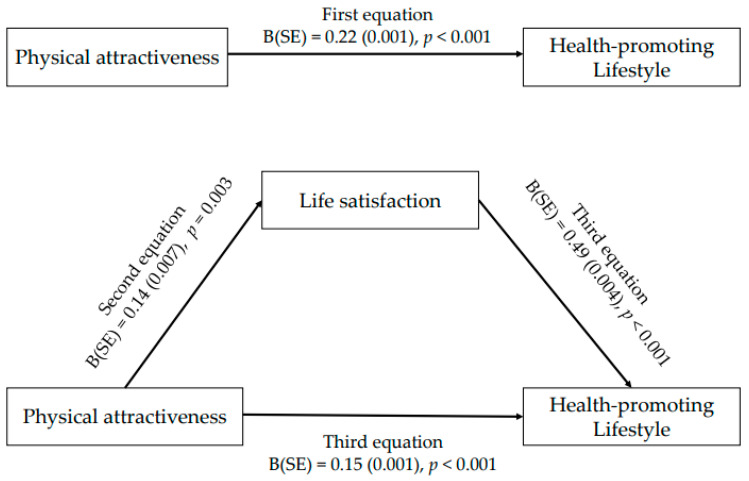
Testing for a mediating effect of life satisfaction on the association between physical attractiveness and health-promoting lifestyle.

Based on the study purpose, we hypothesized as follows:

1. Perceived physical attractiveness, life satisfaction, and lifestyle behaviors will be associated with each other.
2. The higher life satisfaction will be associated with the greater adherence to health-promoting lifestyle behaviors.
3. Life satisfaction will mediate the relationship between perceived physical attractiveness and lifestyle behaviors of health (Figure 1).

## 2. Methods

### 2.1. Participants and Procedure

The study participants were 459 adults aged over 18 years old in Seoul and Gyeonggi province of Korea through convenience sampling. We explained the study purpose and method to 540 people at supermarkets, cultural centers, and community centers, and 500 people agreed to participate in the survey, but we used 459 data for the final analysis, excluding missing and incomplete responses. We classified the participants into a young adults group (n = 215) and a middle-aged and older adults group (n = 244) based on age 45 [18]. We confirmed the sample size using the G*Power 3.1 program, taking into account six independent variables based on the median effect size f^2^ = 0.15, significance level (α) 0.05, power (1−β) 95% of the linear regression analysis. The number of study participants met the sample size criteria of at least 146 people. We surveyed from May to October 2019. Participants took 10–15 min to complete the survey. 

### 2.2. Measures

#### 2.2.1. Sociodemographic Variables

We collected four sociodemographic variables: age, gender, educational level, and job status in a short answer format.

#### 2.2.2. Perceived Physical Attractiveness

The participants self-rated on a 100-point scale from “0 = not attractive at all” to “100 = perfectly attractive” for their physical attractiveness.

#### 2.2.3. Life Satisfaction

We assessed life satisfaction using the Satisfaction with Life Scale, developed by Diner et al., a 5-item, 7 Likert scale that ranges from 7, strongly agree, to 1, strongly disagree. [19]. This widely validated scale measures life satisfaction as an antecedent of the judgmental component of subjective well-being [11,19]. According to psychological classification, judgmental, evaluative subjective well-being corresponds to life satisfaction [20]. A total score of more than 21 points indicates satisfaction with life.

The scale was validated in a Korean population study, and the reliability, represented with Cronbach’s alpha, was 0.89. [21]. Cronbach’s alpha of this study was 0.84.

#### 2.2.4. Lifestyle Behaviors of Health

We measured lifestyle behaviors of health with 17 items of health-promoting behaviors in the LOHAS scale developed by Soo-Yeon Choi [22]. It is a five-point Likert scale and consists of 5 domains. “LOHAS” is an acronym for the Lifestyle of Health and Sustainability. LOHAS, as an evolved concept, means not only one’s health but also a sustainable consumption base and environment. In “LOHAS”, health-promoting behaviors mean making an effort to reach an optimal state in all areas: physical, emotional, social, spiritual, and intellectual domains related to the individual. The physical lifestyle of health (4 items) measures behaviors such as regular exercise, considered food choices, abstinence from harmful materials such as drugs, tobacco, and alcohol, and active participation in the medical check-up. The emotional domain (4 items) measures emotional and stress control behaviors, coping against failures and frustrations, fostering positive thinking, and being passionate about everyday life. The social domain (3 items) measures volunteering for family, friends, colleagues, neighbors, and the community and being meaningful in society. The spiritual lifestyle (3 items) measures having a sense of meaning and purpose in life, sharing feelings of joy, love, and peace with others, and trying to help others in a difficult situation. The intellectual domain (3 items) measures the continuing pursuit of self-development, enjoying new challenges, and actively sharing knowledge or experiences with others. Cronbach’s alpha of this study was 0.90.

### 2.3. Statistical Analysis

We performed all statistical analyses using the SPSS/WIN 23.0 program (IBM, Armonk, NY, USA). Independent *t*-test for continuous variables and chi-square test for categorical variables evaluated differences between young adults and middle-aged and older adults groups. We adopted a partial correlation test to evaluate correlations between crucial variables, adjusting for age, gender, marital status, education, and job status, and linear multiple regression analysis to test a causal relationship. For the mediation effect confirmation, we applied Sobel’s test.

### 2.4. Ethical Considerations

The Internal Review Board approved the research methods and the procedure’s scientific and ethical issues before data collection (IRB number: SHIRB-201809-HR-084-02).

## 3. Results

The average age was 31.6 years for young adults and 55.4 years for middle-aged and older adults (Table 1). The proportions of gender and job status between the groups differed. There was no significant difference between the groups in physical attractiveness and life satisfaction. Life satisfaction scores were 22.5 and 22.7, respectively, and evaluated as slightly satisfied in both groups. The lifestyle of health was lower for young adults with 3.39, compared to 3.66 points for middle-aged and older adults. The middle-aged and older adults showed higher scores in every domain except intellectual lifestyle behaviors compared with young adults (Table 1).

Table 2 shows positive associations among physical attractiveness, life satisfaction, and lifestyle behaviors related to health even though adjusted for age, gender, marital status, education, and job status (*p* < 0.05).

In Table 3, the high life satisfaction group showed higher scores in every domain and total lifestyle of health than the low life satisfaction group did (*p* = 0.001).

As shown in Table 4, there was a positive association of physical attractiveness with the higher lifestyle of health (ß = 0.221, *p* < 0.001) in the first equation. In the second equation, physical attractiveness also exhibited a positive association with higher life satisfaction (ß = 0.141, *p* = 0.003). Furthermore, physical attractiveness continued to exert a direct effect on the lifestyle of health (ß = 0.152, *p* < 0.001), even though the association was slightly eliminated after adjustment for life satisfaction in the third equation, rather than in the first (ß = 0.221, *p* < 0.001). Sobel’s test confirmed a significant mediating effect of life satisfaction (z = 2.80, *p* = 0.005). Life satisfaction partially mediated the relationship between perceived physical attractiveness and a higher lifestyle of health (Figure 1).

## 4. Discussion

This study investigates the mediating effect of life satisfaction on the relation between perceived physical attractiveness and health-promoting lifestyle in Korean adults. There was no difference in the perceived physical attractiveness between the young adults and the middle-aged and older groups when examining the populations’ characteristics. This result indicates that body satisfaction does not decrease with age. It is possible to improve the positive perception of appearance despite the physical changes caused by the aging process. It is based on ideal body adjustment closer to age-appropriate reality and not oriented to comparisons with celebrities.

The middle-aged and older adults showed a higher tendency to engage in healthy lifestyle behavior than young adults did in the study. As people age, they tend to engage in healthy lifestyle behaviors such as medical check-ups, nonsmoking, and healthy eating [23,24]. This tendency was consistent with our study. These results reflect that while young adults are busy carrying out life tasks, middle-aged and older adults tend to lead more stable lives with settled jobs [25]. For this reason, thanks to affordability and time allowance, middle-aged and older adults can more actively engage in health-promoting behaviors and the effects of health threats as they age.

Physical attractiveness is a subjective cognition based on a socio-cultural ideal appearance and interpersonal context, while body image is a self-perception based on individual subjectivity. Most physical attractiveness stereotypes include a thin, muscular, fair, and youthful physical appearance [8,20,26]. However, these attractiveness stereotypes are constructed and transmitted under various social influences such as media, peers, family members, cultures, and social identities [27]. In young Koreans, they tend to evaluate their physical attractiveness by comparing their appearance with Hallyu stars, worldwide Korean media stars such as in drama or music shows, exposed in the media [26]. Additionally, young Koreans tend to put much effort into maintaining their facial beauty or staying skinny, because physical conditions such as beautiful faces or thinness act as an essential factor in determining physical attractiveness and affecting social relationships [26].

The more attractive person tends to be evaluated as more intelligent, cooperative, and competent, and obtain various positive social benefits [27]. It is possible to infer that these positive effects of perceived attractiveness impact life satisfaction. Frederick et al. [28] reported the relationship between weight and appearance satisfaction with life satisfaction. Previous studies also reported a strong relationship between physical attractiveness and psychological well-being [4], especially life satisfaction [29]. Psychologists classified psychological well-being into evaluative, hedonic, and eudemonic well-being, and life satisfaction corresponds to evaluative well-being [30]. This fact is consistent with this study’s result that perceived physical attractiveness showed an association with life satisfaction even after adjusting for various demographic characteristics.

Perceived physical attractiveness is associated with the health-promoting lifestyle behaviors in this study. Some studies reported that individuals with body dissatisfaction or distortion are more likely to engage in unhealthy behaviors such as physical inactivity, more screen time, more alcohol, and cigarette use [20,28,31].

Perceived physical attractiveness tends to make people perform desirable behaviors. Meanwhile, perceived unattractiveness tends to make people engage in undesirable behaviors. Researchers may explain this tendency by the self-fulfilling prophecy theory that beliefs determine behaviors, and these behaviors consequently reinforce and internally verify preexisting expectations [32].

Life satisfaction mediates the relationship between perceived physical attractiveness and lifestyle behaviors of health in this study. In the perspective of positive psychology, perceived physical attractiveness is a positive individual trait (strength). Moreover, life satisfaction has both causal properties for lifestyle behaviors regarding positive subjective experiences and consequential properties for physical attractiveness in evaluative well-being. Unlike existing problem-oriented psychology, positive psychology advantaged personal and social strengths and assets to solve problems [5]. Positive subjective experiences, positive individual traits, and positive interpersonal relationships are associated with rapid wound healing, physiological reserves, and strong disease resistance [5]. Some studies emphasize the importance of life satisfaction in health-promoting behaviors such as physical activity, healthy eating, abstaining from smoking, and sun protection [33,34]. In addition, others reported the associations of life satisfaction with stress coping strategies, which indicate emotional lifestyle of health [35], with social relations [36,37], with life meaning that indicates spiritual lifestyle of health [35,38], and with knowledge-sharing that indicates intellectual lifestyle of health [37]. This study also found that the higher the satisfaction with life, the better the adherence to a health-promoting lifestyle.

There are some limitations to this study. As the research targets Korean adults, careful considerations are needed to interpret and generalize another socio-cultural population. Moreover, the proportion of male participants in this study was small. Therefore, we propose further studies on subjects with similar gender proportions in diverse populations.

We assessed perceived physical attractiveness using a single item because we judged that no tool could accurately measure such an ambiguous concept. It seems necessary to develop tools through conceptual analysis.

Moreover, its cross-sectional nature limits assurance of causal inferences about the mediating effect. The study’s strength is that it reveals the link between perceived physical attractiveness, life satisfaction, and health-promoting lifestyle behaviors after the full adjustment for potential confounding factors for health promotion and disease prevention from a positive psychological perspective. We propose additional longitudinal studies to confirm the relationship between them in various socio-cultural contexts.

## 5. Conclusions

For health promotion and disease prevention, positive psychology can be a suitable strategy in that it uses positive attributes such as strengths and assets to solve problems. So far, body image studies have been directed to emphasize negative perceptions of the body. On the contrary, perceived physical attractiveness, which can act as a strength and asset from a positive psychological perspective, can be an alternative to body image for health promotion and disease prevention research. A systematic review study indicates that interventions intending to promote positive body image affect health and well-being [39]. We suggest that physical attractiveness and life satisfaction can be essential keys when planning nursing interventions to maintain health-promoting behaviors.

## Figures and Tables

**Table 1:** General participant characteristics (N = 459).

Variables	Young Adults (n = 215)	Middle-Aged and Older Adults (n = 244)	*χ*^2^ or *t*	*p*
	Mean ± SD or n (%)			
Age (year)	31.57 ± 8.26	55.40 ± 9.26	−28.94	<0.001
Gender				
Male	19 (8.8)	43 (17.8)	7.84	0.005
Female	196 (91.2)	198 (82.2)		
Education (>high school)	101 (47.0)	104 (43.3)	0.61	0.435
Job status, yes	116 (54.0)	184 (76.7)	26.05	<0.001
Physical attractiveness	70.38 ± 47.79	73.80 ± 14.77	−1.01	0.314
Life satisfaction	22.49 ± 4.91	22.74 ± 4.98	−0.54	0.593
Lifestyle of health, total	3.39 ± 0.60	3.66 ± 0.48	−5.03	<0.001
Physical	3.26 ± 0.71	3.73 ± 0.59	−7.57	<0.001
Emotional	3.61 ± 0.73	3.89 ± 0.57	−4.52	<0.001
Social	2.91 ± 0.81	3.24 ± 0.72	−4.62	<0.001
Spiritual	3.68 ± 0.71	3.81 ± 0.63	−2.04	0.042
Intellectual	3.49 ± 0.78	3.52 ± 0.74	−0.37	0.715

**Table 2:** Partial correlation coefficients for physical attractiveness, life satisfaction, and lifestyle of health (N = 459).

Categories	Variables	Physical Attractiveness	Life Satisfaction	Lifestyle of Health, Total	Physical	Emotional	Social	Spiritual	Intellectual
		r (*p*)							
Young adults									
	Physical attractiveness	1							
	Life satisfaction	0.137 (0.046)	1						
	Lifestyle of health, total	0.238 (<0.001)	0.564 (<.001)	1					
	Physical	0.177 (0.010)	0.395 (<.001)	0.697 (<0.001)	1				
	Emotional	0.233 (0.001)	0.553 (<.001)	0.869 (<0.001)	0.488 (<0.001)	1			
	Social	0.102 (0.140)	0.428 (<.001)	0.814 (<0.001)	0.380 (<0.001)	0.641 (<0.001)	1		
	Spiritual	0.257 (<0.001)	0.500 (<.001)	0.881 (<0.001)	0.530 (<0.001)	0.717 (<0.001)	0.679 (<0.001)	1	
	Intellectual	0.206 (0.003)	0.424 (<.001)	0.837 (<0.001)	0.390 (<0.001)	0.682 (<0.001)	0.669 (<0.001)	0.735 (<0.001)	1
Middle- and old-aged adults									
	Physical attractiveness	1							
	Life satisfaction	0.245 (<0.001)	1						
	Lifestyle of health, total	0.264 (<0.001)	0.496 (<0.001)	1					
	Physical	0.138 (0.036)	0.252 (<0.001)	0.622 (<0.001)	1				
	Emotional	0.161 (0.015)	0.462 (<0.001)	0.777 (<0.001)	0.376 (<0.001)	1			
	Social	0.218 (0.001)	0.352 (<0.001)	0.764 (<0.001)	0.262 (<0.001)	0.487 (<0.001)	1		
	Spiritual	0.237 (<0.001)	0.427 (<0.001)	0.791 (<0.001)	0.294 (<0.001)	0.581 (<0.001)	0.571 (<0.001)	1	
	Intellectual	0.238 (<0.001)	0.364 (<0.001)	0.776 (<0.001)	0.328 (<0.001)	0.446 (<0.001)	0.558 (<0.001)	0.567 (<0.001)	1

Adjusted for age, gender, marital status, education, and job status.

**Table 3:** Comparison of differences in lifestyle behavior according to life satisfaction.

	Low Life Satisfaction (n = 134)	High Life Satisfaction (n = 325)	*t*	*p*
	Mean ± SD			
Lifestyle of health, total	3.16 ± 0.53	3.69 ± 0.49	−10.25	<0.001
Physical	3.22 ± 0.69	3.63 ± 0.65	−5.87	<0.001
Emotional	3.33 ± 0.68	3.94 ± 0.57	−9.86	<0.001
Social	2.71 ± 0.73	3.24 ± 0.74	−7.09	<0.001
Spiritual	3.37 ± 0.67	3.90 ± 0.60	−8.35	<0.001
Intellectual	3.09 ± 0.75	3.68 ± 0.69	−8.07	<0.001

**Table 4:** Hypothesis testing for a mediating effect of life satisfaction on the association between physical attractiveness and lifestyle of health.

Variables	ß	SE	*p*-Value
First equation			
Outcome variable: lifestyle of health			
Independent variable: physical attractiveness	0.221	0.001	<0.001
Second equation			
Outcome variable: life satisfaction			
Independent variable: physical attractiveness	0.141	0.007	0.003
Third equation			
Outcome variable: lifestyle of health			
Mediator: life satisfaction	0.493	0.004	<0.001
Independent variable: physical attractiveness	0.152	0.001	<0.001
Sobel’s test, z = 2.80, *p* = 0.005			

ß = standardized regression coefficient; SE = standard error. All multiple regression models were adjusted for age, sex, education level, and job status.
